# A Complementary Dual-Branch Receptive Field Network for Cross-Channel-Density Motor Imagery EEG Decoding

**DOI:** 10.3390/biomimetics11100690

**Published:** 2026-09-23

**Authors:** Mengnan Zhu, Kendi Li, Junbiao Zhu, Yantao Chen, Hui Cao, Jing Xiao, Zuguang Rao

**Affiliations:** 1 School of Artificial Intelligence, Guangzhou Maritime University, Guangzhou 510725, China; mengnanzhu1013@163.com (M.Z.); caohui@gzmtu.edu.cn (H.C.); xiaojing@gzmtu.edu.cn (J.X.); 2 School of Artificial Intelligence, South China Normal University, Guangzhou 510631, China; likendi@m.scnu.edu.cn; 3 School of Electronic and Information Engineering, Huaihua University, Huaihua 418000, China; junbiaozhu01@163.com; 4 School of Automation Science and Engineering, South China University of Technology, Guangzhou 510640, China; 202510183685@mail.scut.edu.cn

**Keywords:** brain–computer interface (BCI), motor imagery (MI), dual-branch, multi-scale, cross-channel-density

## Abstract

Brain–computer interfaces (BCIs) enable direct communication between the brain and external devices, providing a bio-inspired link between humans and artificial systems. However, electroencephalography (EEG)-based motor imagery (MI) decoding continues to pose challenges, due to limited temporal exploitation and insufficient spatio-temporal modeling. To alleviate these limitations, a complementary dual-branch receptive field network (DBRFNet) is proposed for cross-channel-density MI-EEG decoding. Specifically, two complementary branches with distinct temporal receptive fields are designed to capture multi-scale temporal dynamics. Each branch employs a progressive spatio-temporal module integrating temporal, depthwise spatial, and dilated convolutions for modeling short- and long-range temporal dependencies. The features from the two branches are fused using element-wise addition and further encoded for classification. The proposed method is evaluated on three public datasets representing low-, medium-, and high-density EEG configurations. DBRFNet achieves decoding accuracies of 80.48%, 84.37%, and 92.24% under cross-validation analysis, and 87.76%, 80.94%, and 94.64% under hold-out analysis, demonstrating competitive performance compared with state-of-the-art methods. Ablation studies further validate the effectiveness of each component within the proposed architecture. These results indicate that DBRFNet effectively learns discriminative spatio-temporal representations and provides a robust solution for MI-BCI systems.

## 1. Introduction

Brain–computer interface (BCI) systems enable direct neural information transfer between the brain and external systems [1]. Compared with other neural recording techniques, electroencephalography (EEG) has been widely adopted owing to its non-invasive, low cost, and high temporal resolution [2,3]. As a key BCI paradigm, motor imagery (MI) involves the mental simulation of actions in the absence of overt motor output [4]. In MI scenarios, the sensorimotor cortex exhibits distinct neural oscillations, manifesting as event-related desynchronization/synchronization (ERD/ERS) [5]. Reliable decoding of these EEG patterns enables effective interaction with external devices and shows great potential in assistive control [6], neurorehabilitation [7], and post-stroke motor function recovery [8]. Therefore, improving the reliability and effectiveness of MI-EEG decoding is essential for translating BCIs into practical applications.

Reliable MI-EEG decoding is challenged by non-stationary signals, noise artifacts, and large inter-subject variability [9]. Among conventional machine learning methods for MI-EEG analysis, common spatial pattern (CSP) is widely used to extract features via spatial filters that maximize inter-class variance for enhanced discriminability [10]. Based on CSP, several extensions have been proposed, including filter-bank common spatial pattern (FBCSP) [11], probabilistic CSP [12] and regularized CSP [13], to further improve feature discriminability. Following feature extraction, baseline models such as support vector machines (SVM) or linear discriminant analysis (LDA) is commonly used due to their low computational cost and competitive performance [14]. However, the effectiveness of these conventional methods relies on manual feature extraction, which requires substantial task-specific expertise [15]. Moreover, they are limited in learning complex nonlinear representations directly from raw EEG signals.

Deep learning models have emerged as effective alternatives, enabling the automatic extraction of high-dimensional and nonlinear EEG features. For example, Schirrmeister et al. [16] proposed Shallow and Deep ConvNets for end-to-end EEG classification, with performance comparable to conventional FBCSP without manual feature engineering. By integrating depthwise separable convolutions, EEGNet proposed by Lawhern et al. [17] significantly reduces parameter complexity while maintaining competitive decoding performance. Recent deep learning studies have expanded along multiple directions, such as temporal modeling for long-range dependency extraction [18], graph-based methods for modeling brain topological relationships [19], hybrid convolution-attention models for joint local-global feature learning [20], and metric learning approaches for improving feature discriminability [21]. Despite recent advancements, MI decoding still suffers from limited temporal representation when using a single temporal receptive field. Moreover, the optimal receptive field for temporal dynamics is highly variable across subjects and recording protocols due to cross-subject and cross-session variability [22]. These characteristics motivate the use of complementary temporal representations at different scales rather than relying on a single fixed temporal scale.

Multi-branch architectures employ parallel filter banks with multi-scale temporal windows, capturing both short- and long-range temporal dependencies. For instance, a filter-bank-inspired convolutional architecture was proposed in [23], which decomposes MI-EEG signals into multiple frequency bands and processes them through parallel streams to enhance feature representation. To extract cross-scale temporal dynamics, the multi-scale Vision Transformer leverages self-attention mechanisms to model sequence dependencies across different resolution granularities [4]. In addition, several studies have incorporated multi-scale mechanisms into self-designed convolutional models, such as a multi-domain CNN with MixConv-Residual blocks [24] and a deep multi-scale attentional CNN with feature-fusion attention [25]. These approaches consistently demonstrate the effectiveness of multi-scale representation learning for MI-EEG decoding. However, existing multi-scale networks often rely on multiple parallel branches, which may introduce feature redundancy and additional computational requirements.

To reduce feature redundancy while preserving complementary information, some studies have adopted dual-branch architectures. For instance, IFNet employs two frequency bands (4–16 Hz and 16–40 Hz) to extract frequency-specific representations [26]. DBConformer employs two parallel conformer-based branches to model temporal and spatial features, respectively [27], while Dual-TSST adopts a dual-branch Transformer architecture to process raw EEG and wavelet-transformed time-frequency representations [28]. These methods construct complementary representations from frequency, spatial-temporal, or input-domain perspectives. However, the explicit use of distinct temporal receptive fields to construct complementary temporal representations remains less explored. Although Transformer-based models can effectively capture long-range dependencies through self-attention, their weak locality inductive bias and reliance on sufficient training data may limit their suitability for MI-EEG decoding [29]. Convolutional architectures, in contrast, provide strong local inductive biases and efficient temporal feature extraction, making them attractive for MI-EEG decoding with limited training data.

Motivated by these considerations, we propose a dual-branch receptive field network (DBRFNet) for MI-EEG decoding. Unlike existing dual-branch methods that construct complementary representations mainly from frequency, input-domain, or spatial-temporal perspectives, DBRFNet adopts distinct temporal receptive fields to capture relatively long-range and fine-grained temporal characteristics, respectively. Each branch integrates temporal, depthwise spatial, and dilated temporal convolutions for progressive spatio-temporal feature learning, and the two branches are fused through element-wise addition. By combining distinct temporal receptive fields with progressive spatio-temporal feature learning, DBRFNet provides a different mechanism for constructing complementary multi-scale representations from existing dual-branch approaches. The contributions are summarized as follows:

- We propose a dual-branch receptive field architecture that explicitly assigns distinct temporal receptive fields to two complementary branches for multi-scale temporal representation learning.
- We design a progressive spatio-temporal feature learning framework that integrates temporal, depthwise spatial, and dilated temporal convolutions to jointly model temporal dynamics and inter-channel spatial dependencies.
- We conduct extensive evaluations and ablation studies on three public MI-EEG datasets with low-, medium-, and high-density EEG configurations, demonstrating the effectiveness and cross-channel-density robustness of DBRFNet.

## 2. Methodology

As shown in Figure 1, the proposed DBRFNet consists of two parallel temporal-spatial CNN branches (Branch I and Branch II) and a classification module. To effectively exploit multi-scale temporal and spatial information, a dual-branch architecture is designed, where convolutional kernels with different receptive fields are used to capture complementary EEG characteristics. Temporal convolutions with larger kernel sizes capture broader frequency components, while smaller kernels focus on higher-frequency dynamics [30]. Spatial convolutions are applied to extract discriminative spatial patterns across EEG channels [23]. The outputs of the two branches are fused via feature addition, which aligns feature representations and reduces redundancy while maintaining a compact representation. Finally, a fully connected layer is used for classification. The source code for DBRFNet is available at https://github.com/zuguangrao/DBRFNet (accessed on 18 September 2026).

For clarity, we denote a single EEG trial as $x=R^{F\times C\times T}$, with its corresponding label $y\in [0,1,\ldots ,N_{c}-1]$. Here, F, C, and T represent the feature dimension, number of EEG channels, and number of temporal samples, respectively, while $N_{c}\;$ denotes the number of classes. In our implementation, the input feature dimension is set to F=1. The architecture of DBRFNet is detailed in the following section.

In the first CNN branch (Branch I), the model is designed to capture fine-grained spatio-temporal features. Specifically, $F_{1}$ temporal convolutional kernels of size $1\times K_{11}$ are first applied to the input EEG signals to extract high-frequency components, as defined in Equation (1), followed by batch normalization to stabilize feature distributions. A second temporal convolutional layer with the same kernel size and $F_{2}$ output channels is then applied to further enhance temporal representations, as described in Equation (2), also followed by batch normalization. Next, a depthwise separable spatial convolution is used to model inter-channel relationships. This layer employs $F_{2}\times M$ kernels of size C×1 to capture spatial dependencies across EEG channels, where *M* denotes the depth expansion factor and C  is the number of channels, as formulated in Equation (3). A max-norm constraint with a threshold of 1 is imposed on the convolutional weights to improve training stability and enhance the physiological interpretability of the learned spatial features. The above operations can be formulated as follows:

(1) $$X_{T}^{\prime F_{1}}=Conv_{\left(1\times K_{11}\right)}(X^{\prime }),$$

(2) $$X_{T}^{\prime F_{2}}=Conv_{\left(1\times K_{11}\right)}(X_{T}^{\prime F_{1}}),$$

(3) $$X_{S}^{\prime F_{2}\times M}=DWConv_{\left(C\times 1\right)}(X_{T}^{\prime F_{2}}),$$

where $X^{\prime }$ denotes the input EEG signal, $X_{T}^{\prime F_{1}}$ and $X_{T}^{\prime F_{2}}$ represent the outputs of the temporal convolution layers, and $X_{S}^{\prime F_{2}\times M}$ denotes the output of the spatial convolution layer. Finally, batch normalization, ELU activation, average pooling with kernel size $P_{1}$, and dropout with rate $P_{c}$ are sequentially applied for nonlinear transformation and dimensionality reduction.

To enlarge the temporal receptive field, two dilated temporal convolutional layers with kernel size $1\times K_{12}$ are introduced, with dilation rates $D_{11}$ and $D_{12}$, where $D_{12}>D_{11}$ to progressively expand the receptive field, as defined in Equations (4) and (5). By leveraging parameter-efficient dilated convolutions, this design expands the receptive field with minimal computational overhead, enabling the modeling of long-range temporal dependencies and multi-scale temporal patterns. Finally, a $1\times P_{2}$ kernel average pooling layer compresses the output tensor. The dilated temporal convolution operations can be formulated as follows:

(4) $$X_{T_{1}}^{\prime F_{2}}=Conv_{\left(1\times K_{12}\right)}^{D_{11}}(X_{P_{1}}^{\prime }),$$

(5) $$X_{T_{1}}^{\prime F_{1}}=Conv_{\left(1\times K_{12}\right)}^{D_{12}}(X_{T_{1}}^{\prime F_{2}}),$$

where $T_{1}$ denotes the temporal length after average pooling with kernel size $P_{1}$, and $X_{T_{1}}^{\prime F_{2}}$ and $X_{T_{1}}^{\prime F_{1}}$ denote the outputs of the dilated convolutional layers with dilation rates $D_{11}$ and $D_{12}$, respectively. 

In the second CNN branch (Branch II), the model is designed to capture large-scale temporal structures and low-frequency components. While sharing the same overall architecture as Branch I, it differs in kernel sizes $\left(K_{21},\;K_{22}\right)$ and dilation rates $\left(D_{21},\;D_{22}\right)$. This configuration enables complementary temporal modeling across different scales, enhancing the model’s multi-scale representation capability.

To fully integrate features from different-scale branches, an element-wise addition strategy is adopted for feature fusion, as defined in Equation (6). This preserves the feature dimensionality while aligning multi-scale representations and reducing redundancy. The fused features are then normalized using Layer Normalization to stabilize their distribution, followed by GELU activation and dropout (rate $P_{c}$) to improve nonlinearity and alleviate overfitting. Finally, a fully connected layer with a max-norm constraint (0.25) is used as the classifier to produce the final predictions, as formulated in Equation (7). The fusion and classification process can be formulated as follows:

(6) $$X_{T_{2}}^{\prime F_{2}}=X_{T_{2}}^{\prime F_{1}}+X_{T_{2}}^{\prime \prime F_{1}},$$

(7) $$\hat{y}=\mathrm{Softmax}(WX_{T_{2}}^{F_{1}}+b),$$

where $T_{2}$ denotes the temporal length after average pooling with kernel size $P_{2}$, $X_{T_{2}}^{F_{1}}$ represents the fused features obtained via element-wise addition, W and b denote the weight matrix and bias vector of the classifier, respectively, and $\hat{y}$ is the predicted probability distribution over all classes. 

## 3. Evaluations

### 3.1. Dataset Description

To evaluate the effectiveness of the proposed DBRFNet, we conducted evaluations on three publicly available and widely used offline MI-EEG datasets, namely BCIC-IV-2b, BCIC-IV-2a, and HGD, representing low-, medium-, and high-density EEG configurations, respectively. These datasets have been extensively used as benchmark datasets in previous MI-EEG decoding studies, providing standardized and reproducible evaluation settings [19,28]. More importantly, their substantially different EEG channel densities enable a systematic assessment of DBRFNet under varying channel configurations. The key characteristics and preprocessing procedures of the three datasets are summarized in Table 1.

**BCIC-IV-2b** [11], a low-density EEG dataset, employs a tri-electrode configuration (C3, Cz, and C4) sampled at 250 Hz for binary MI classification (left-hand vs. right-hand) across five sessions per subject. The first three sessions serve as the training set, while the last two are used for testing, resulting in approximately 400 training and 320 testing trials. Each trial is segmented within a 3–7 s post-cue interval, producing a 1000-point temporal sequence.

**BCIC-IV-2a** [31], a medium-density EEG dataset, comprises EEG data from nine subjects performing four MI tasks (left hand, right hand, feet, and tongue). EEG signals are recorded using 22 channels at 250 Hz. Each subject performed two sessions with 288 trials per session (72 per class). Each trial is segmented within a 2–6 s interval, producing 1000 samples per trial.

**HGD** [16], a high-density EEG dataset, includes EEG data from 14 subjects performing a four-class MI task. The data were originally recorded using 128 channels at 500 Hz, and we follow the benchmark setting in [16], by down sampling to 250 Hz and selecting 44 channels. Each subject has approximately 880 training and 160 testing trials, with each trial segmented within a 0–4 s post-stimulus window, resulting in 1000 samples per trial.

For the BCIC-IV-2a and BCIC-IV-2b datasets, we adopted the original preprocessing pipeline provided by the dataset authors, which includes a band-pass filter of 0.5–100 Hz and a 50 Hz notch filter to remove power-line noise [32]. For the HGD dataset, a third-order Butterworth high-pass filter with a cutoff frequency of 4 Hz was applied [19]. After the respective preprocessing procedures, no additional normalization or standardization was applied to the EEG signals from any of the three datasets before they were input to the network.

### 3.2. Comparison Methods

The benchmark methods are summarized as follows:

(1) **Shallow ConvNet** [16] is designed for oscillatory signal classification and employs two convolutional architectures for temporal and spatial feature learning.

(2) **EEGNet** [17] is a lightweight model that applies temporal, depthwise spatial, and pointwise convolutions for feature extraction.

(3) **FBCNet** [23], inspired by the FBCSP algorithm [11], adopts a multi-band filter-bank convolutional architecture for spectral–spatial EEG feature learning.

(4) **CTNet** [33] is a CNN-Transformer framework for MI-EEG classification, capturing both local and global temporal dependencies.

(5) **FBLightConvNet** [34] is a lightweight CNN that integrates temporal modeling with attention mechanisms to capture spatio-temporal features.

(6) **FBMSNet** [35] integrates filter banks, multi-scale temporal convolutions, spatial filtering, and log-variance feature learning for MI-EEG feature extraction.

(7) **IFNet** [26] is designed for MI-EEG decoding via a three-stage framework consisting of spectro-spatial representation learning, cross-frequency interaction modeling, and classification.

(8) **MSVTNet** [4] is a multi-scale Vision Transformer-based model that captures multi-scale temporal and spatial representations via parallel convolutional branches and Transformer-based feature aggregation.

### 3.3. Evaluation Setups

Both cross-validation (CV) and hold-out (HO) analyses were conducted to evaluate model generalization [25]. For the CV analysis, Session 1 of BCIC-IV-2a and the training sets of BCIC-IV-2b and HGD were used for model training. A five-fold class-balanced cross-validation scheme was adopted, with one fold used for testing and the others for training. For the HO analysis, Session 1 and Session 2 of BCIC-IV-2a were used for training and testing, respectively, while BCIC-IV-2b and HGD followed their predefined training–testing splits. 

To configure the hyperparameters of DBRFNet, HO analysis was first performed using the first session of the BCIC-IV-2a dataset, as shown in Table 2. The second session was reserved exclusively as the final test set and was not involved in hyperparameter tuning. The selected hyperparameter configuration was then fixed and applied to all other datasets without further hyperparameter tuning, following the protocol in [4]. For fair comparison, all benchmark methods adopted the hyperparameter settings reported in their original publications. Additionally, performance was evaluated using accuracy (ACC) and F1-score (F1). Statistical significance was tested via the Wilcoxon signed-rank test, and multiple comparisons were corrected using the two-stage Benjamini–Hochberg false discovery rate procedure (FDR-TSBH) [5].

### 3.4. Training Details

In this study, the model was trained using the cross-entropy loss function and optimized with the Adam optimizer at a learning rate of 0.001. Considering the limited availability of EEG data, a two-stage training strategy [23], was adopted to fully utilize the data. Specifically, the training data were partitioned into training and validation subsets, with one fold used for validation under CV and 20% of the training data reserved for validation under the HO setting. In the first stage, training was monitored using validation accuracy and terminated if no improvement was observed for 200 consecutive epochs or when the maximum of 1500 epochs was reached. At the subsequent stage, training was continued from the model checkpoint with the highest validation accuracy obtained in the first stage. The process was terminated when the validation loss fell below the minimum training loss from the first stage or when the number of epochs reached 600.

## 4. Results and Discussion

### 4.1. Performance Comparison

Table 3 shows the comparative decoding accuracies and F1-scores obtained by different methods under both CV and HO analyses on the three datasets. Under the CV analysis, DBRFNet achieved the best performance on both the BCIC-IV-2a and 2b datasets, with accuracies of 84.37% and 80.48%, respectively, and corresponding F1-scores of 84.06% and 80.37%. Compared with the highest baseline methods, DBRFNet improved decoding accuracy by 5.76% and 1.85% on BCIC-IV-2a and 2b, respectively. For HGD dataset, DBRFNet achieved an accuracy of 92.24% and an F1-score of 92.22%, ranking second with performance comparable to that of the best-performing.

Under the more challenging HO analysis, DBRFNet demonstrated even greater advantages. Specifically, it achieved the highest accuracies of 80.94%, 87.76%, and 94.64% on the three datasets, with corresponding F1-scores of 80.71%, 87.73%, and 94.56%. Compared with the second-best methods, DBRFNet improved accuracy by 3.66%, 2.85%, and 1.25% on the three datasets, respectively, indicating superior generalization to unseen test samples.

Statistical analyses further validated the effectiveness of DBRFNet. Significant improvements over most competing methods were observed across all three datasets, particularly under the HO analysis, even after multiple-comparison correction. Moreover, the performance gains of DBRFNet were consistently reflected in both ACC and F1, indicating improved overall classification accuracy as well as a better balance between precision and recall. Overall, these results demonstrate that DBRFNet can effectively learn discriminative spatio-temporal representations from MI-EEG signals and achieve robust performance across different datasets.

Figure 2 presents the number of subject-level evaluations with decoding accuracy exceeding 70%. The 70% threshold is commonly used as a reference criterion for effective binary BCI control [23,35]. For consistency, the same 70% threshold was adopted for both binary and four-class MI-BCI to assess BCI usability in this study. For each subject, one evaluation was obtained from the CV analysis and another from the HO analysis, resulting in two evaluations per subject. These two evaluations correspond to the same subject rather than to independent subjects. It can be seen that the proposed DBRFNet consistently obtained the largest number of evaluations across all datasets. Specifically, DBRFNet achieved 18 out of 18 successful evaluations on the BCIC-IV-2a dataset, indicating that all subject-level evaluations satisfied the usability criterion. On the BCIC-IV-2b dataset, 17 out of 18 evaluations surpassed the threshold, outperforming all competing methods. Similarly, on the HGD dataset, DBRFNet achieved 28 out of 28 successful evaluations, demonstrating highly reliable decoding performance. These findings indicate that the proposed method not only enhances average decoding performance but also boosts the proportion of subjects achieving over 70% accuracy, improving the usability of the MI-BCI system.

### 4.2. Confusion Matrices

Figure 3 shows the confusion matrices of the top five performing methods. For the BCIC-IV-2a dataset, all compared methods achieved relatively high accuracies along the diagonal entries. Nevertheless, misclassifications were still observed between certain classes, particularly between the foot and tongue tasks. Compared with these approaches, DBRFNet achieved higher diagonal values in most classes, demonstrating enhanced class-wise discrimination in multiclass MI-EEG decoding. On the BCIC-IV-2b dataset, all methods achieved relatively high classification accuracy. Among them, DBRFNet exhibited the most balanced performance, with diagonal accuracies of 88.2% and 87.7% for the two classes, respectively, resulting in the smallest inter-class discrepancy. For the HGD dataset, DBRFNet achieved recognition accuracies exceeding 93% across all classes, with consistently low off-diagonal values, indicating effective discrimination among different MI categories. Overall, the proposed model showed stronger class-wise discriminative capability, as evidenced by higher diagonal values and fewer misclassifications.

### 4.3. t-SNE Visualization

To further evaluate the learned representations, t-SNE visualizations of classification-layer embeddings were generated for the three datasets, as illustrated in Figure 4. The subjects were selected based on their relatively high test accuracy achieved by DBRFNet in the hold-out (HO) analysis, with one subject selected from each dataset. For each selected subject, the corresponding held-out test-data embeddings were visualized to examine the feature representations learned by the trained model. The preprocessed EEG trials were directly fed into the trained networks, and the feature representations before the final classification layer were extracted and flattened into feature vectors. No additional PSD extraction, normalization, or standardization was performed. The resulting high-dimensional feature vectors were then directly projected into a two-dimensional space using t-SNE, with a perplexity of 30, a learning rate of 200, and PCA initialization. Here, PCA was used only for initialization within t-SNE and was not applied as a separate preprocessing or dimensionality-reduction step. On BCIC-IV-2a, DBRFNet generated more compact clusters and clearer class boundaries than the competing methods, indicating better separation between classes in four-class MI decoding. On BCIC-IV-2b, DBRFNet exhibited more concentrated and well-separated distributions, suggesting enhanced robustness and stable feature learning. Similarly, on the HGD dataset, DBRFNet showed clearly separated clustering patterns with minimal inter-class overlap. Overall, these t-SNE visualizations provide qualitative evidence of the discriminative and well-separated feature representations learned by DBRFNet for the selected subjects.

### 4.4. Ablation Study

Table 4 presents the ablation results of DBRFNet on the three datasets. Specifically, Branch1 and Branch2 correspond to the two branches of the proposed dual-branch framework. “w/o Dilation” denotes the variant where the dilation operation is removed by setting all dilation rates to 1. “w/o MultiScale (25)” and “w/o MultiScale (75)” denote variants without multi-scale temporal modeling, where both branches use identical kernel sizes at a fixed temporal scale (i.e., *k*_11_ = *k*_21_ = 25 and *k*_11_ = *k*_21_ = 75, respectively).

On the BCIC-IV-2a dataset, Branch1 and Branch2 achieved accuracies of 81.56% and 78.74%, respectively, indicating different contributions from the two temporal branches. Removing dilated convolution led to a decrease in classification accuracy, indicating that enlarged receptive fields are beneficial for modeling long-range temporal dependencies. The w/o MultiScale (25) variant achieved the highest accuracy (82.37%), while w/o MultiScale (75) achieved only 76.39%, indicating that the effectiveness of a fixed temporal scale depends on dataset characteristics. Although w/o MultiScale (25) slightly outperformed DBRFNet (80.94%), this result suggests that a single temporal scale can be advantageous for this particular dataset.

On the BCIC-IV-2b dataset, all variants achieved comparable performance due to the relatively lower complexity of the binary classification task. Nevertheless, DBRFNet still achieved the highest accuracy, demonstrating the complementary effects of the proposed components. On the HGD dataset, DBRFNet achieved the best performance (94.64%), while removing either dilated convolution or multi-scale processing resulted in performance degradation. In particular, DBRFNet outperformed Branch1 and Branch2 by 3.75 and 5.76 percentage points, respectively, demonstrating the benefit of combining complementary temporal representations.

Overall, although certain components show dataset-specific advantages, the complete DBRFNet consistently achieves competitive performance across datasets. The results indicate that the effectiveness of temporal scales is dataset-dependent, while complementary receptive fields can provide more robust spatio-temporal representations across different MI-EEG datasets.

### 4.5. Computational Complexity Analysis

To quantitatively evaluate the computational requirements of DBRFNet and the comparison methods, three metrics were considered: the number of trainable parameters, floating-point operations (FLOPs), and inference time. The inference time was measured with a batch size of 1 to simulate real-time decoding of a single input sample. Each model was first subjected to 10 warm-up forward passes, followed by 100 consecutive forward passes, and the average elapsed time was used as the inference time. All inference-time measurements were performed on an NVIDIA GeForce RTX 2060 GPU.

As shown in Table 5, DBRFNet contains 22.56 K trainable parameters, requires 41.03 M FLOPs, and achieves an average inference time of 1.69 ms per input sample. Although DBRFNet does not have the lowest number of parameters or FLOPs among all comparison methods, its inference latency remains relatively low. Compared with FBCNet, FBMSNet, and MSVTNet, DBRFNet achieves lower inference latency. Notably, DBRFNet employs only two complementary branches with distinct temporal receptive fields, whereas these methods use multiple parallel branches for frequency-specific or multi-scale representation learning. Rather than increasing the number of branches to enhance multi-scale representation capability, DBRFNet utilizes two branches with different temporal receptive fields to capture fine-grained temporal information and long-range temporal dependencies, respectively, achieving a good balance between multi-scale temporal representation and computational efficiency. Although the current study focuses on algorithm-level performance evaluation, the architecture of DBRFNet is composed primarily of standard convolutional and lightweight spatial operations, which provides a feasible basis for subsequent edge-oriented implementation.

### 4.6. Parameter Sensitivity Analysis

To investigate the effects of the numbers of *F*_1_ and *F*_2_ filters on the performance of the proposed method, a parameter sensitivity analysis was performed on the three datasets. Specifically, *F*_1_ was set to 6, 9, 12, and 18, while *F*_2_ was set to 12, 24, 36, and 48. All other network and training settings were kept unchanged. 

As shown in Figure 5a, the model performance generally improves as the numbers of temporal filters (*F*_1_) and spatial filters (*F*_2_) increase. A larger filter scale enables the extraction of richer temporal-frequency features and spatial information, enhancing the representational capacity of the model. The highest accuracy of 82.64% was achieved with *F*_1_ = 18 and *F*_2_ = 48. However, the performance gain gradually exhibits a diminishing-return effect as the filter scale increases. For example, the accuracy difference between the configurations *F*_1_ = 18, *F*_2_ = 36 and *F*_1_ = 18, *F*_2_ = 48 is only 0.43%. This suggests that although increasing the number of filters can strengthen feature representation, the resulting performance improvement becomes increasingly limited. Therefore, it is necessary to further determine an appropriate parameter configuration by jointly considering the performance on the other two datasets.

On the BCI-IV-2b dataset (Figure 5b), the performance differences among various parameter configurations are relatively small, with classification accuracies consistently ranging from 87% to 88%, indicating that the model is relatively robust to changes in filter numbers. Unlike the trend observed in Figure 5a, increasing the filter scale does not lead to continuous performance improvement. For example, when *F*_1_ = 18, the accuracy decreases from 87.50% to 87.01% as *F*_2_ increases from 24 to 48. The highest accuracy of 88.25% is achieved with *F*_1_ = 12 and *F*_2_ = 24. These results suggest that, for datasets with relatively lower task complexity, a moderate filter scale is sufficient to capture discriminative EEG representations. Further increasing the number of filters may introduce redundant features and unnecessary model complexity, resulting in performance saturation or even slight degradation, as similarly reported in [4].

The results on the HGD dataset (Figure 5c) exhibit a different pattern from those observed on the previous two datasets. The model is more sensitive to the number of spatial filters (*F*_2_). For instance, with *F*_1_ = 9, increasing *F*_2_ from 12 to 36 improves the classification accuracy from 91.74% to 94.64%. However, further increasing *F*_2_ to 48 results in performance saturation and even slight degradation in some cases. This suggests that a moderate increase in the number of spatial filters enables the model to learn richer and more discriminative spatial representations associated with MI activities. Nevertheless, excessively large spatial filter scales may introduce redundant information, limiting further performance gains [30]. In contrast, temporal filter numbers (*F*_1_) ranging from 9 to 18 consistently achieve strong performance, indicating that the model is relatively robust to variations in *F*_1_.

To determine a unified parameter configuration, the average classification accuracy across the three datasets was further analyzed. As shown in Figure 5d, the model performance generally improves as the numbers of *F*_1_ and *F*_2_ increase. By considering classification performance and model complexity, *F*_1_ = 9 and *F*_2_ = 36 were selected as the default configuration. This setting achieved the highest average accuracy (87.78%) while exhibiting strong stability and generalization capability across different datasets.

### 4.7. Multi-Branch Analysis

To determine the optimal number of branches in DBRFNet, different branch configurations were evaluated on the BCIC-IV-2a dataset. Specifically, the *K*_1_ kernel configurations for the two-branch (proposed), three-branch, and four-branch architectures were set to [25, 75], [25, 50, 75], and [15, 25, 50, 75], respectively. Smaller kernels were designed to capture local short-term temporal patterns, whereas larger kernels were used to model long-range temporal dependencies [24]. Correspondingly, the *D*_1_ and *D*_2_ dilation rate configurations were set to [3, 2] and [4, 3] for the two-branch (proposed) architecture, [4, 3, 2] and [5, 4, 3] for the three-branch architecture, and [5, 4, 3, 2] and [6, 5, 4, 3] for the four-branch architecture. All other network parameters were kept unchanged.

As shown in Figure 6, the performance differences between the proposed model (dual-branch) and the three-branch and four-branch models are not statistically significant. Specifically, when the number of branches increases from two to three or four, no further performance improvement is observed, and a slight decline is even seen on some datasets. This finding suggests that, although increasing the number of branches can introduce richer temporal-scale representations, it may also lead to feature redundancy and increased model complexity, weakening the complementarity and discriminative ability among branches [36]. In contrast, the dual-branch architecture better balances short-term local features and long-term temporal dynamics, achieving higher decoding accuracy with fewer branches. Therefore, the dual-branch structure is adopted as it achieves a better trade-off between classification performance and model complexity.

### 4.8. Explainability Analysis

To further investigate the spatial representations learned by DBRFNet, the spatial convolution weights were visualized and analyzed for all nine subjects in the BCIC-IV-2a dataset. As shown in Figure 7, the most sensitive channels in both branches are primarily distributed around the sensorimotor cortex, particularly near C1, Cz, and C2. These regions are closely associated with MI tasks and are the primary cortical sites where ERD/ERS phenomena occur [37]. The activations of these channels suggest that the proposed model can effectively capture discriminative spatial patterns related to MI tasks.

In addition, it can be observed that the two branches exhibit distinct spatial distributions. Specifically, the first branch produces relatively smooth and widespread weight distributions (e.g., Subjects 3, 4, 7 and 8), enabling the extraction of more global spatial features. In contrast, the second branch demonstrates stronger local selectivity, with weights concentrated on a small number of key channels (e.g., Subjects 1, 5, 6, 7 and 8), facilitating the learning of local spatial patterns. These observations suggest that the two branches learn complementary spatial representations at different scales rather than redundant information. Overall, the proposed dual-branch architecture focuses on MI-related cortical regions and learns complementary global and local spatial features. These patterns are consistent with neurophysiological findings in MI, highlighting the model’s interpretability. 

### 4.9. Limitations and Future Work

Despite its promising performance, the proposed approach still has some limitations. First, the proposed method was mainly evaluated using CV and HO analyses on public datasets, and its generalization ability in more challenging scenarios, such as cross-subject settings [38], still requires further investigation. Future studies will focus on domain adaptation [39] or transfer learning [40] to enhance the robustness of the proposed model. Second, trial augmentation has been widely recognized as an effective strategy for improving decoding performance [41], and future studies will explore more advanced EEG data augmentation methods. Third, the receptive field configurations are currently determined empirically, while the optimal temporal scale may vary across datasets with different characteristics. Developing adaptive receptive field mechanisms [42] that can automatically adjust temporal scales according to data characteristics will be an important direction. Fourth, further validation on larger and more diverse datasets and investigation of the neurophysiological relevance of the learned EEG representations will be pursued in future work. Finally, the current study does not include FPGA/ASIC implementation or hardware-level validation. Future work will investigate hardware resource utilization, power consumption, and device-level inference latency to assess the feasibility of DBRFNet for real-time edge BCI deployment.

## 5. Conclusions

This study presents DBRFNet for MI-EEG decoding, featuring a dual-branch architecture with complementary temporal receptive fields and a progressive spatio-temporal feature learning strategy to capture both local and long-range dependencies. The proposed method is evaluated on three public datasets (low-, medium-, and high-density), achieving decoding accuracies of 80.48%, 84.37%, and 92.24% under CV analysis, and 87.76%, 80.94%, and 94.64% under HO analysis. It achieves better performance than state-of-the-art methods in most cases, with ablation studies confirming each component’s effectiveness. These findings indicate that DBRFNet can effectively capture discriminative spatio-temporal features, offering a reliable solution for MI-BCI decoding.

## Figures and Tables

**Figure 1:**
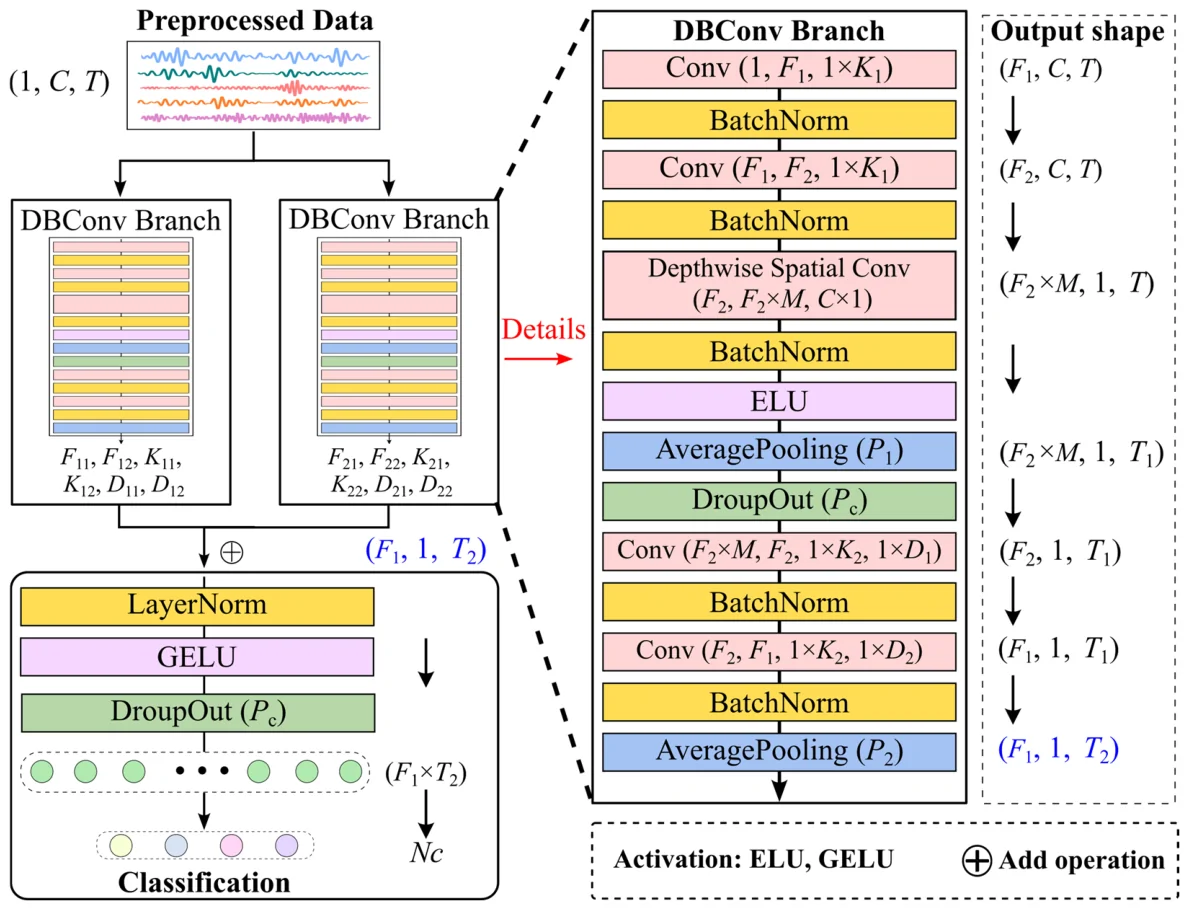
Overall architecture and processing pipeline of the proposed DBRFNet.

**Figure 2:**
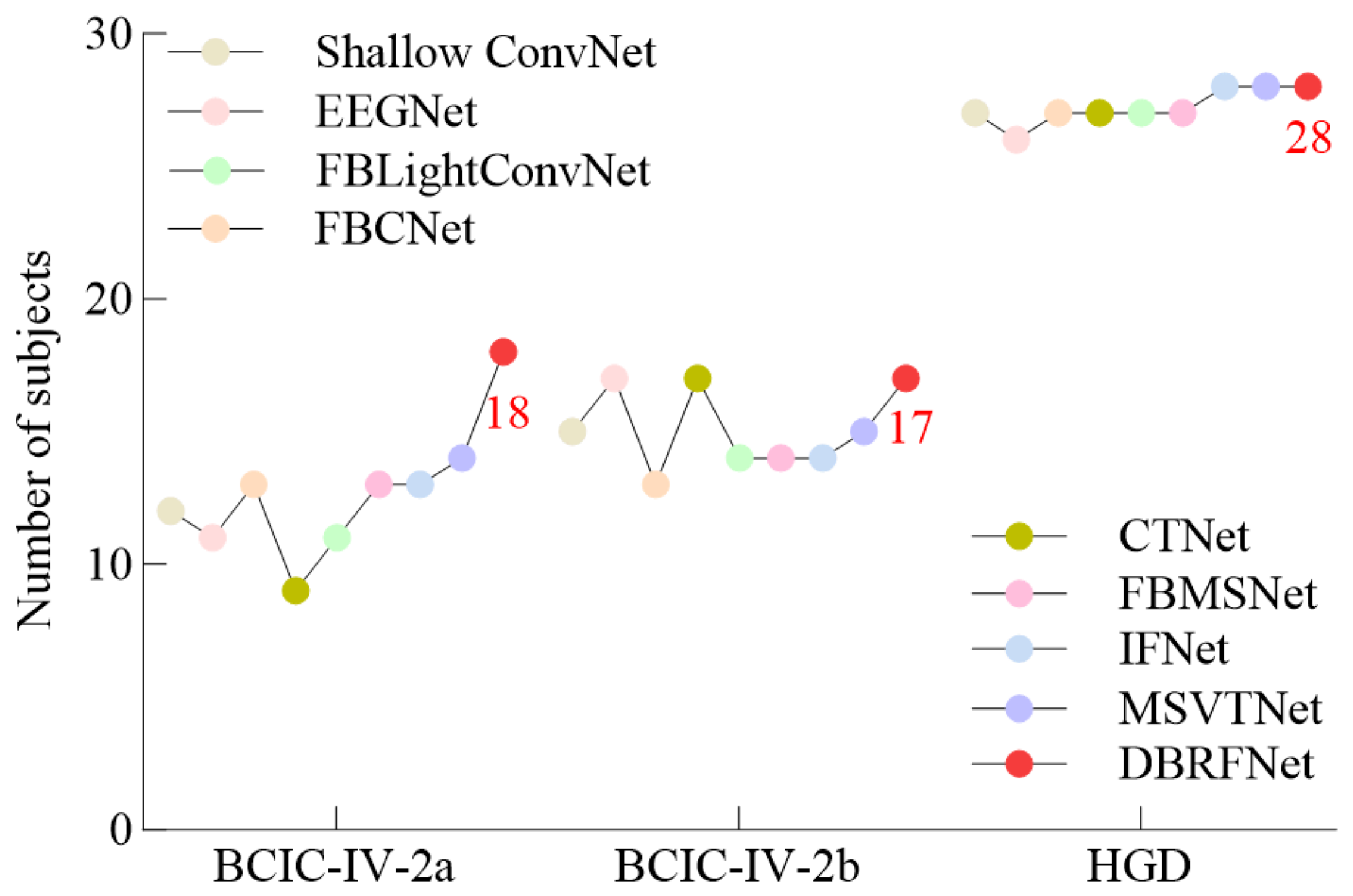
Number of subject-level evaluations achieving decoding accuracies above 70% on the three datasets. The counts were obtained by adding the results from both CV and HO evaluations, resulting in a total of 17, 18, and 28 subject-level evaluations for the three datasets, respectively.

**Figure 3:**
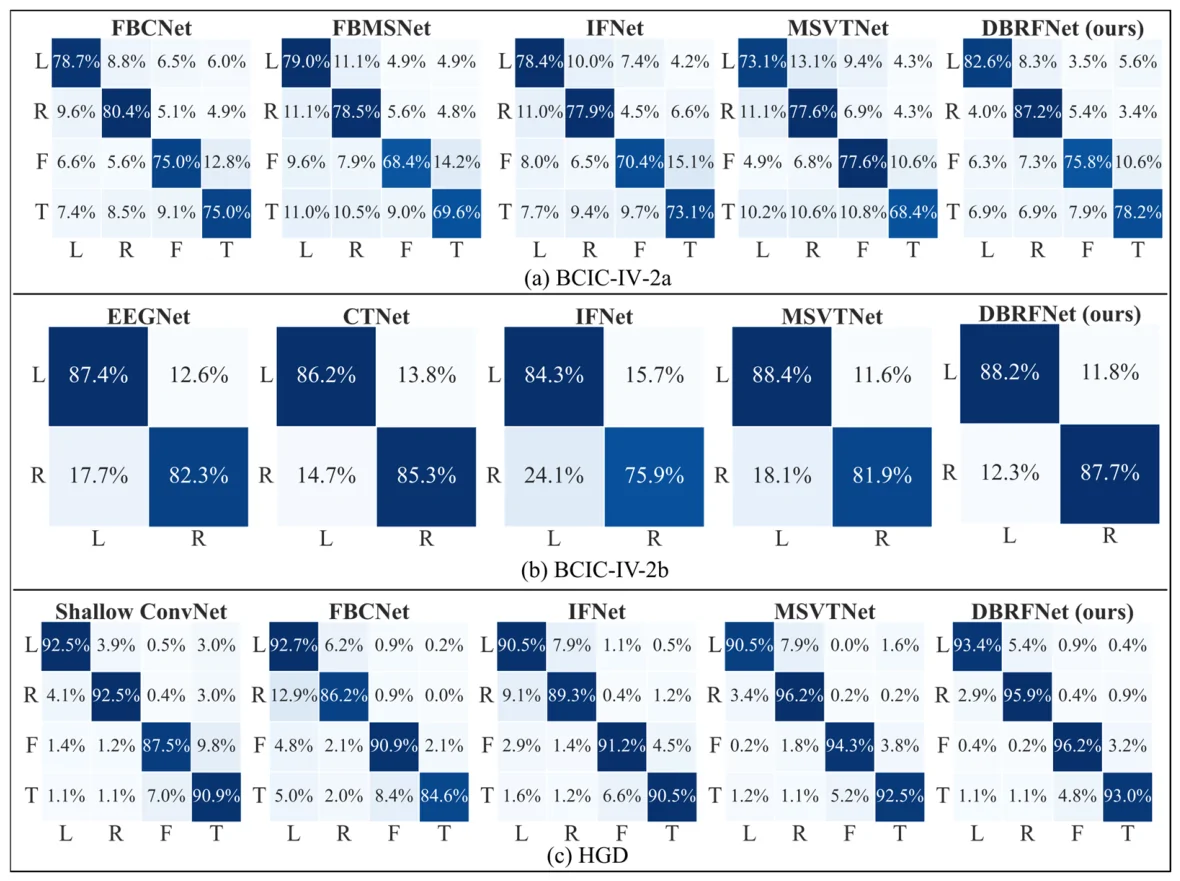
Confusion matrices (%) of the top five performing methods on the three datasets. Rows and columns denote the predicted and true classes, respectively. L, R, F, and T represent the left hand, right hand, feet, and tongue, respectively. The dark blue color indicates higher classification accuracy, while lighter colors indicate lower classification accuracy.

**Figure 4:**
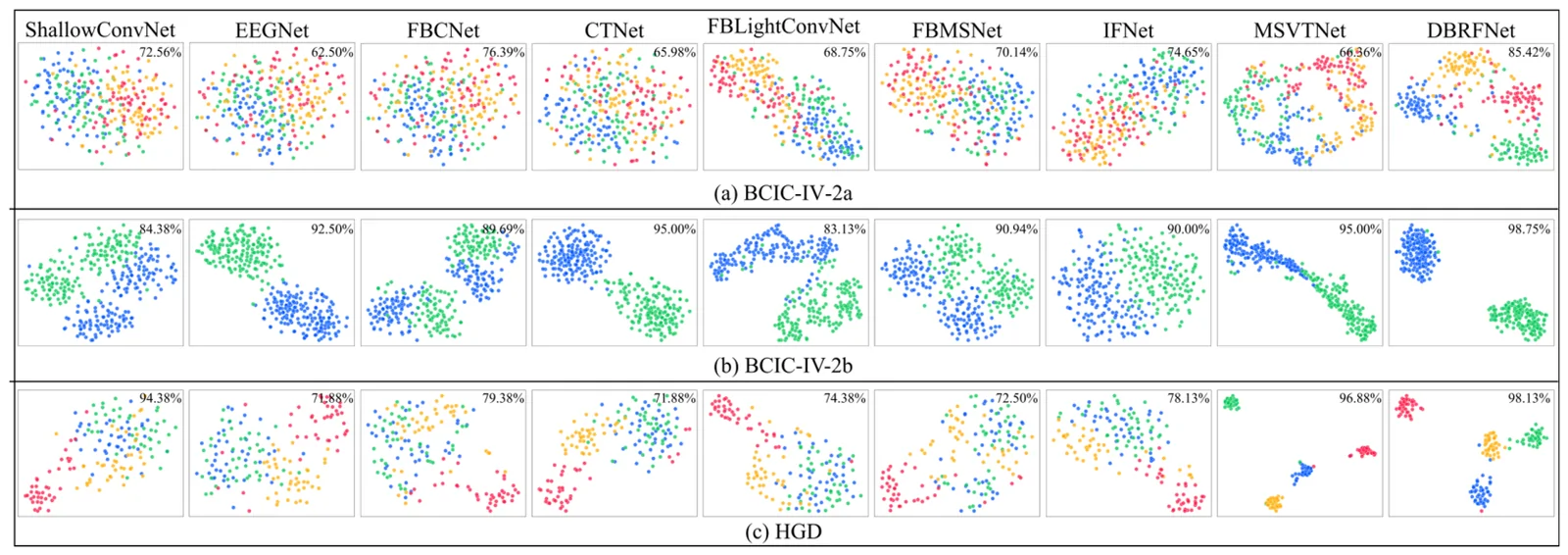
t-SNE visualization of feature embeddings extracted from the held-out test data at the classification layer in the HO analysis for Subject 4 on BCIC-IV-2a, Subject 5 on BCIC-IV-2b, and Subject 11 on the HGD dataset. Each point represents one EEG trial, and different colors indicate different MI classes. The accuracy value shown in the upper-right corner of each subfigure represents the test-set accuracy of the corresponding subject in the HO analysis.

**Figure 5:**
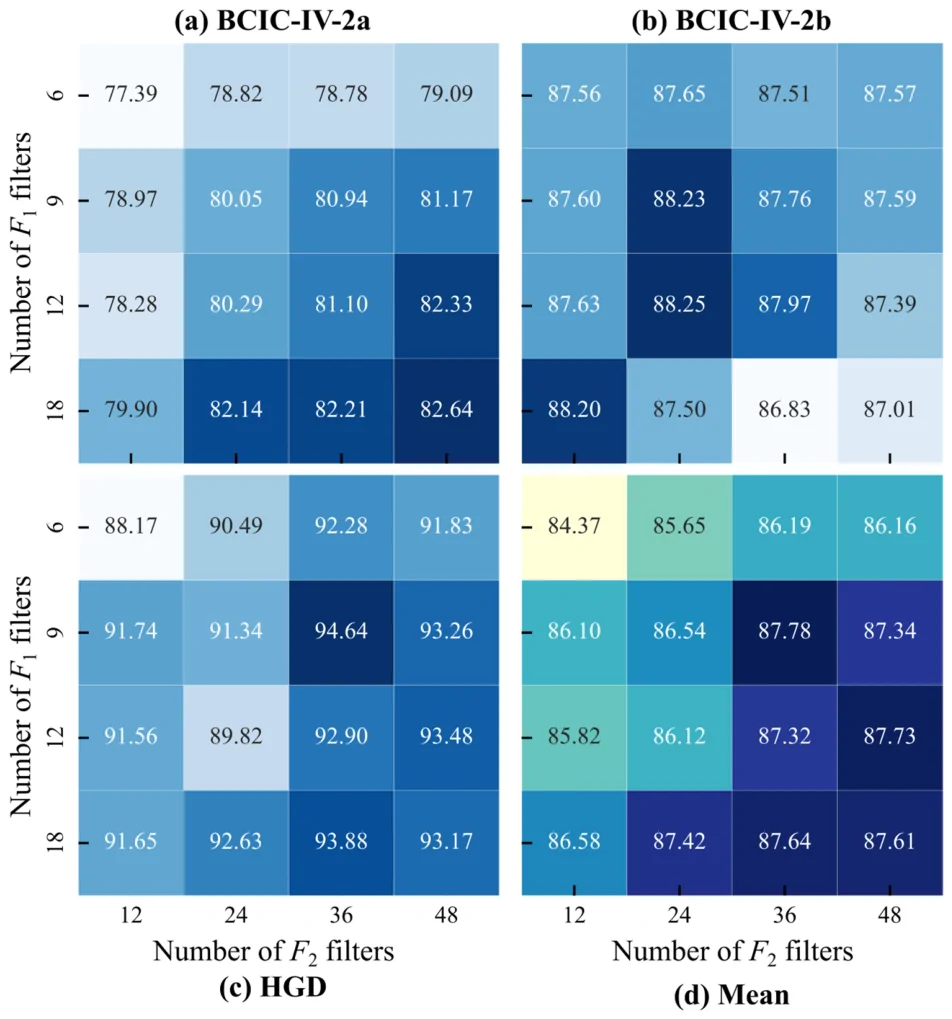
Parameter sensitivity analysis of the numbers of F1 and F2 filters. Decoding accuracies (%) achieved under different filter configurations on (**a**) BCI-IV-2a, (**b**) BCI-IV-2b, and (**c**) HGD datasets, as well as (**d**) the mean accuracy across all datasets. The dark blue color indicates higher classification accuracy, while lighter colors indicate lower classification accuracy.

**Figure 6:**
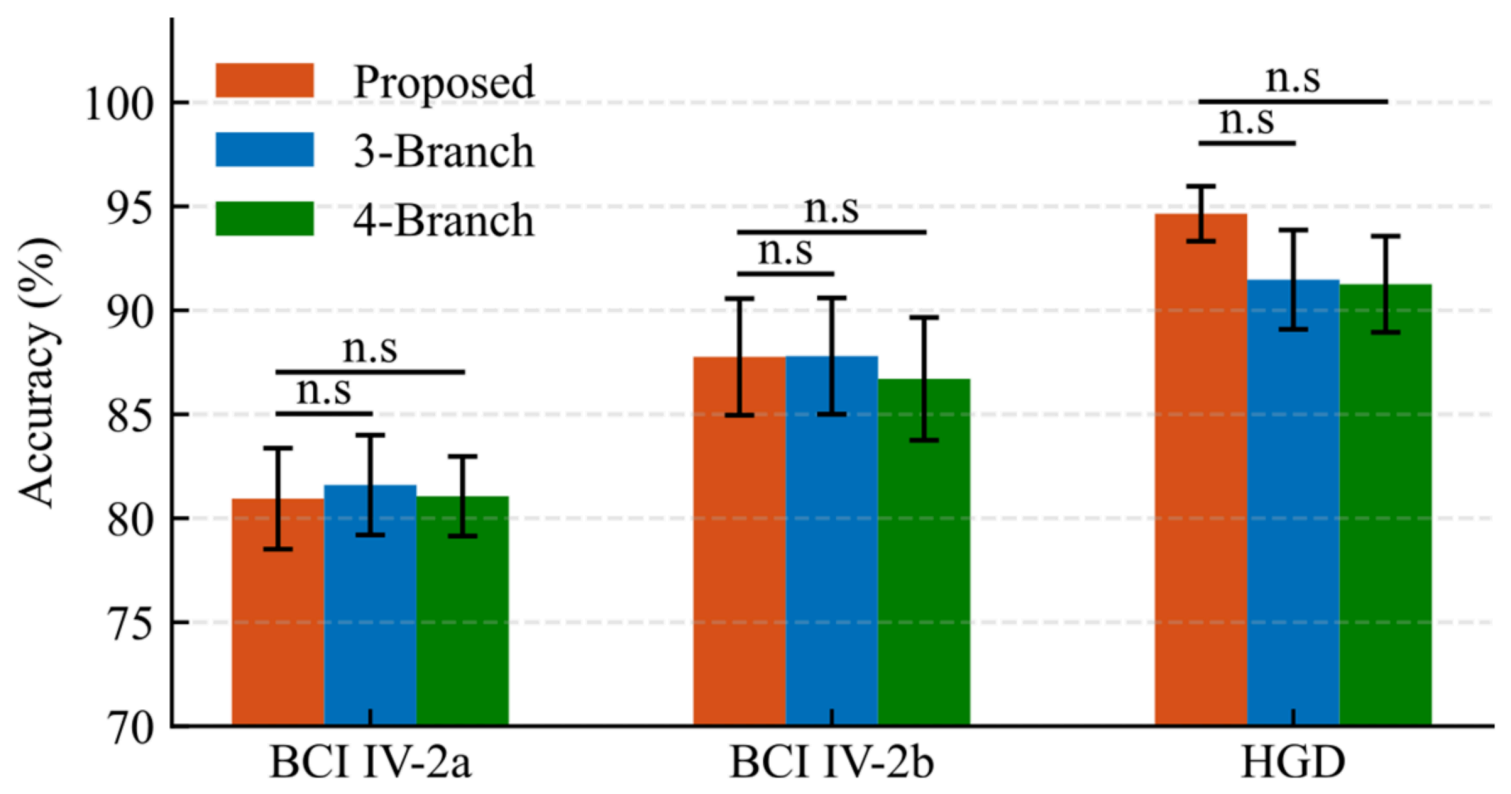
Comparison of classification accuracy among models with different numbers of branches across three EEG datasets. n.s indicates no statistical significance.

**Figure 7:**
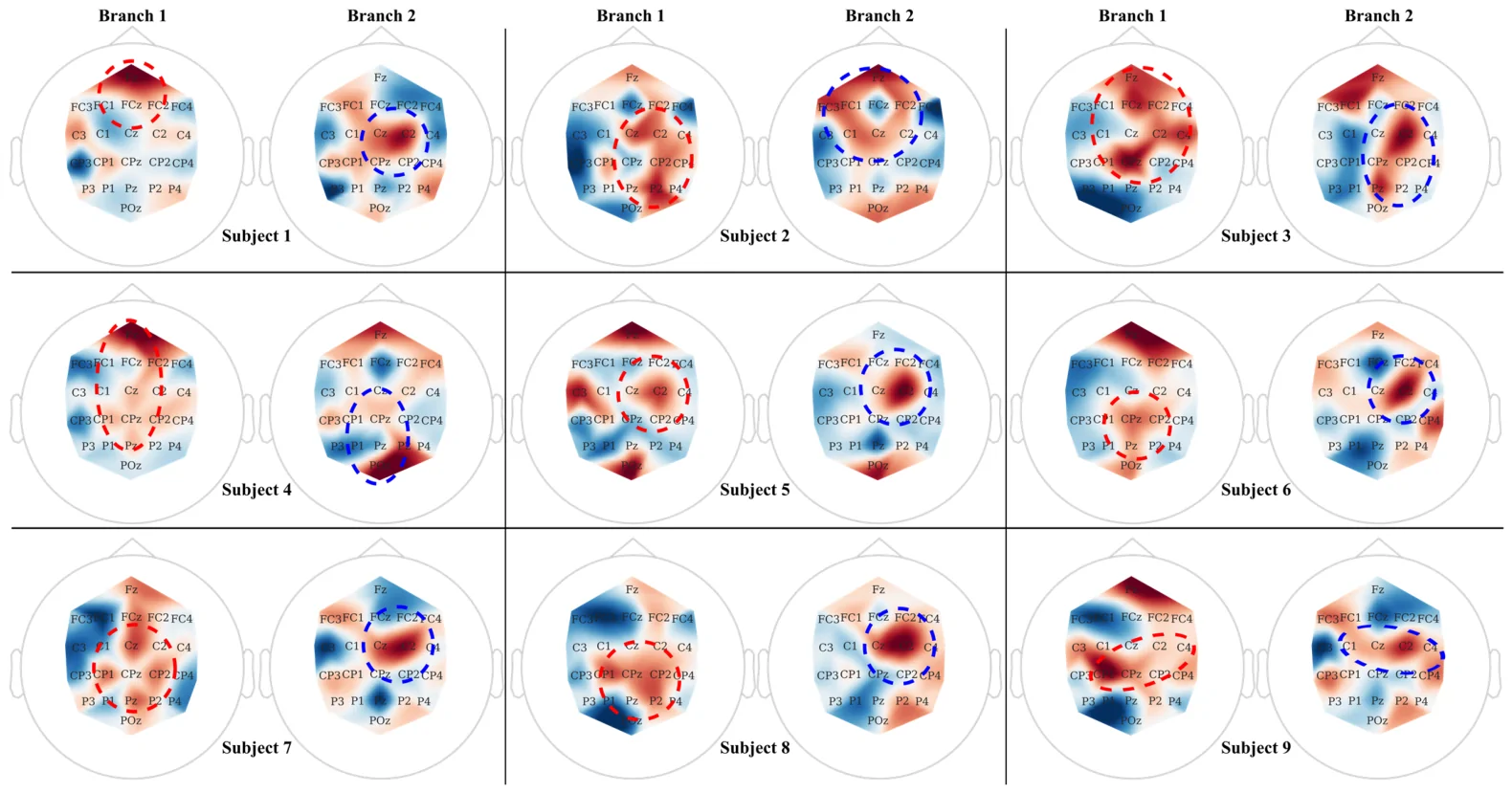
EEG topographic maps learned by the spatial convolution kernels in Branch1 and Branch2 for Subjects 1–9 (evaluated on the first fold of the BCIC-IV-2a dataset using HO analysis). The red and blue dashed circles highlight the dominant spatial activation regions and corresponding electrodes associated with Branch1 and Branch2, respectively.

**Table 1:** Summary of the three MI-EEG datasets used in this study.

Dataset	Density	Subjects	Channels	Sampling Rate	Classes	Trial Segment	Preprocessing
BCIC-IV-2b	Low	9	3	250 Hz	2	3–7 s	0.5–100 Hz band-pass + 50 Hz notch
BCIC-IV-2a	Medium	9	22	250 Hz	4	2–6 s	0.5–100 Hz band-pass + 50 Hz notch
HGD	High	14	44	250 Hz	4	0–4 s	4 Hz third-order Butterworth high-pass

**Table 2:** Hyperparameter settings of Branch I and Branch II modules.

Hyperparameter	Branch I	Branch II
Kernel size	$\left(K_{11},{\;K}_{12})=(25,\;15\right)$	$\left(K_{21},\;K_{22})=(75,\;15\right)$
Dilation rates	$\left(D_{11},\;D_{12})=(3,\;4\right)$	$\left(D_{21},{\;D}_{22})=(2,\;3\right)$
Filters $\left(F_{1};\;F_{2}\right)$	$F_{11}=F_{21}=9$; $F_{12}=F_{22}=36$	
Pooling size	$\left(P_{1},{\;P}_{2})=(4,\;8\right)$	
Depth factor	M=2	
Dropout rate	$P_{c}=0.5$	

**Table 3:** Comparative decoding accuracies and F1-scores (mean ± std, %) under CV and HO analyses.

Analyses	Methods	Datasets					
		BCIC-IV-2a		BCIC-IV-2b		HGD	
		ACC	F1	ACC	F1	ACC	F1
CV	Shallow ConvNet	75.02 ± 8.70 *	74.14 ± 10.12 **	75.02 ± 8.70 **	74.75 ± 8.73 **	90.00 ± 7.33	90.00 ± 7.31
	EEGNet	72.91 ± 10.31 **	72.51 ± 10.55 **	78.63 ± 8.20 *	78.41 ± 8.31 *	87.31 ± 7.78 *	87.23 ± 7.85 *
	FBCNet	78.61 ± 12.38	78.26 ± 12.60 *	73.59 ± 11.20 **	73.34 ± 11.34 **	91.47 ± 6.04	91.44 ± 6.09
	CTNet	71.82 ± 9.03 **	71.38 ± 9.37 **	77.67 ± 8.36 **	77.53 ± 8.40 **	84.61 ± 8.80 **	84.57 ± 8.85 **
	FBLightConvNet	77.61 ± 12.71 *	77.19 ± 12.98 **	75.70 ± 12.39 *	75.59 ± 12.40 *	90.18 ± 6.66	90.17 ± 6.67
	FBMSNet	73.64 ± 13.65	75.69 ± 15.33 **	73.64 ± 13.65 **	73.44 ± 13.73 **	89.74 ± 7.08	89.73 ± 7.12
	IFNet	76.17 ± 12.44	75.57 ± 14.66 **	76.17 ± 12.44 *	75.92 ± 12.59 *	90.18 ± 4.97	91.47 ± 5.99
	MSVTNet	77.87 ± 8.90	75.89 ± 11.18 **	77.87 ± 8.90 **	77.67 ± 8.99 **	**92.70 ± 4.97**	**92.67 ± 5.03**
	DBRFNet (ours)	**84.37 ± 7.26**	**84.06 ± 7.64**	**80.48 ± 9.01**	**80.37 ± 9.07**	92.24 ± 6.31	92.22 ± 6.33
HO	Shallow ConvNet	72.07 ± 11.79 **	71.65 ± 11.81 **	79.39 ± 7.40 **	79.12 ± 7.60 **	90.85 ± 8.33 **	90.87 ± 8.13 **
	EEGNet	71.76 ± 12.19 **	71.52 ± 12.27 **	84.61 ± 8.64 **	84.36 ± 8.98 **	86.70 ±10.09 **	86.54 ± 10.14 **
	FBCNet	77.28 ± 10.94 *	76.72 ± 11.34 *	78.86 ± 15.22 **	78.45 ± 15.70 **	88.62 ± 10.47 **	88.68 ± 10.21 **
	CTNet	69.91 ± 7.70 **	69.58 ± 7.86 **	85.53 ± 8.31 **	85.48 ± 8.37 **	85.80 ± 8.96 **	85.85 ± 8.83 **
	FBLightConvNet	72.26 ± 12.41 **	71.38 ± 13.03 **	79.31 ± 15.16 **	78.99 ± 15.44 *	86.96 ± 10.04 **	86.94 ± 9.96 **
	FBMSNet	73.88 ± 13.64 **	73.17 ± 14.17 **	78.64 ± 15.30 **	78.45 ± 15.39 **	87.46 ± 10.42 **	87.50 ± 10.24 **
	IFNet	74.96 ± 13.02 **	74.70 ± 13.17 **	79.79 ± 13.81 **	79.52 ± 13.99 **	90.40 ± 8.13 *	90.25 ± 8.34 **
	MSVTNet	74.19 ± 11.09 **	73.74 ± 11.60 **	84.91 ± 10.38 **	84.45 ± 11.17 **	93.39 ± 5.42 *	93.27 ± 5.64 **
	DBRFNet (ours)	**80.94 ± 6.87**	**80.71 ± 6.98**	**87.76 ± 7.93**	**87.73 ± 7.96**	**94.64 ± 4.76**	**94.56 ± 4.93**

The symbols * and ** indicate that DBRFNet achieves significantly higher decoding performance than the compared methods at *p* < 0.05 and *p* < 0.01, respectively. The best decoding accuracies are highlighted in bold.

**Table 4:** Ablation results of DBRFNet obtained from HO analyses.

Dataset	Method	Accuracy
BCIC-IV-2a	Branch1	81.56 ± 6.00
	Branch2	78.74 ± 7.40
	w/o Dilation	79.71 ± 8.23
	w/o MultiScale (25)	**82.37 ± 7.28**
	w/o MultiScale (75)	76.39 ± 7.61
	DBRFNet	80.94 ± 6.87
BCIC-IV-2b	Branch1	87.19 ± 7.72
	Branch2	87.00 ± 8.72
	w/o Dilation	87.64 ± 7.54
	w/o MultiScale (25)	87.52 ± 7.82
	w/o MultiScale (75)	87.15 ± 7.71
	DBRFNet	**87.76 ± 7.93**
HGD	Branch1	90.89 ± 9.28
	Branch2	88.88 ± 8.89
	w/o Dilation	93.08 ± 6.67
	w/o MultiScale (25)	90.54 ± 9.44
	w/o MultiScale (75)	89.38 ± 8.24
	DBRFNet	**94.64 ± 4.76**

The best decoding accuracies are highlighted in bold.

**Table 5:** Computational complexity and inference latency of all compared methods (k: KILO-10^3^, M: MEGA-10^6^).

Methods	Parameters (k)	FLOPs (M)	Inference Time (ms)
Shallow ConvNet	23.44	163.40	0.70
EEGNet	1.88	11.93	0.82
FBCNet	11.81	1.15	3.77
CTNet	152.68	35.95	7.97
FBLightConvNet	6.60	6.46	3.34
FBMSNet	16.23	99.95	3.70
IFNet	11.40	9.86	1.62
MSVTNet	75.49	51.96	4.63
DBRFNet (ours)	22.56	41.03	1.69

## Data Availability

Publicly available datasets were analyzed in this study. The BCIC-IV 2a and 2b datasets can be found at http://www.bbci.de/competition/iv/ (accessed on 18 September 2026). The HGD dataset is available at https://github.com/robintibor/high-gamma-dataset (accessed on 18 September 2026).
